# P05.21. Kangarooing in German neonatology departments: results of a nationwide survey

**DOI:** 10.1186/1472-6882-12-S1-P381

**Published:** 2012-06-12

**Authors:** M Thiel, A Längler, T Ostermann

**Affiliations:** 1 Univ of Witten/Herdecke, Center Integrative Med, Dept of Pediatrics, Herdecke, Germany; 2 University of Witten/Herdecke, Faculty of Health, School of Medicine, Herdecke, Germany

## Purpose

Kangaroo care is a technique practiced on newborn infants wherein the infant is held, skin-to-skin, with an adult. Our purpose was to assess practical aspects of performing kangaroo-care in German neonatal units.

## Methods

A semi-structured questionnaire has been sent to all pediatric departments with neonatological units in Germany as published in the German hospital list and the German Society of Neonatology (GNPI). After 6 weeks a reminder was sent by e-mail.

## Results

Of the 323 eligible neonatal care units, 162 (50.1%) participated and 160 (98.8%) reported using KC. Instructions for the staff are provided in 39 (25.2%) units, 16 (10.3%) for parents and 64 (41.4%) have hygienical regulations for parents. Special chairs are provided in 143 (89.4%) units, 29 (85.3%) use music, 7 (20.6%) light and 2 (5.9%) aromatherapy as complementary methods. Fifty-one (31.5%) provide security precautions, 22 (26.8%) of them a transcutanous O_2_/CO_2_ probe, 15 (18.3%) a limited number of infants at the same time, and 20 (24.4%) require a physician in close distance. A certain gestational age in 146 units (91.25%) and 142 (88.7%) a certain bodyweight are considered as limits. One hundred thirty-eight (86.25%) have a limitation of days of life before KC. In 63 (38.9%) the gestational age and 69 (42.6%) the birth weight had no influence on first KC. Fifty-two (32.1%) use KC no matter of the infants’ age. Thirty-five (21.6%) departments have other preconditions, where cranial ultrasound is the most common (n=27, 45%).

## Conclusion

This is the first survey on practical aspects of kangaroo-care in Germany. Most neonatal units provide hygienical regulations for staff and parents. Special equipment such as KC-chairs is quite common as well as adding music as a complementary feature. Limitations regarding birth weight and gestational age are more common than other precautions. In conclusion the results could be helpful for clinical studies, especially to describe and compare the used setting or to design a joint concept if multicentre studies need comparable conditions.

